# Key takeaways from Stanford’s symposium on AI for Data Science

**DOI:** 10.1017/cts.2025.10154

**Published:** 2025-09-25

**Authors:** Manisha Desai, John Auerbach, Laurence Baker, Jade Benjamin-Chung, Melissa Bondy, Mary Boulos, Bryan J. Bunning, Ni Deng, Steven N. Goodman, Ivor Horn, Eleni Linos, Mark A. Musen, Lee Sanders, Nigam Shah, Sara Singer, Michelle Williams, James Zou, Michael Pencina

**Affiliations:** 1 Quantitative Sciences Unit, Biostatistics Section, Department of Medicine, Stanford University School of Medicine, Stanford, CA, USA; 2 ICF International, Reston, VA, USA; 3 Department of Health Policy, Stanford University School of Medicine, Stanford, CA, USA; 4 Department of Epidemiology and Population Health, Stanford University School of Medicine, Stanford, CA, USA; 5 Department of Biomedical Data Science, Stanford University School of Medicine, Stanford, CA, USA; 6 Board of Trustees, Boston Children’s Hospital, Boston, MA, USA; 7 Department of Medicine, Stanford University School of Medicine, Stanford, CA, USA; 8 Department of Dermatology, Stanford University School of Medicine, Stanford, CA, USA; 9 Center for Digital Health, Stanford University School of Medicine, Stanford, CA, USA; 10 Center for Biomedical Informatics Research, Department of Medicine, Stanford University School of Medicine, Stanford, CA, USA; 11 Department of Pediatrics, Stanford University School of Medicine, Stanford, CA, USA; 12 Clinical Excellence Research Center, Stanford University School of Medicine, Stanford, CA, USA; 13 Technology and Digital Solutions, Stanford Health Care, Palo Alto, CA, USA; 14 Department of Biostatistics and Bioinformatics, Duke University School of Medicine, Durham, NC, USA

**Keywords:** Artificial intelligence, data science, rigor and reproducibility, education and training, public health

## Abstract

Numerous symposia and conferences have been held to discuss the promise of Artificial Intelligence (AI). Many center on its potential to transform fields like health and medicine, law, education, business, and more. Further, while many AI-focused events include those data scientists involved in developing foundational models, to our knowledge, there has been little attention on AI’s role for data science and the data scientist. In a new symposium series with its inaugural debut in December 2024 titled *AI for Data Science*, thought leaders convened to discuss both the promises and challenges of integrating AI into the workflows of data scientists. A keynote address by Michael Pencina from Duke University together with contributions from three panels covered a wide range of topics including rigor, reproducibility, the training of current and future data scientists, and the potential of AI’s integration in public health.

## Introduction

In today’s rapidly evolving technological landscape, Artificial Intelligence (AI) is undoubtably the most discussed topic. Broadly, AI can be defined as the ability of a computer system to perform tasks that typically require human intelligence, such as learning, reasoning, and making decisions [1]. Similarly, the Encyclopedia Britannica defines AI as “the ability of a digital computer or computer-controlled robot to perform tasks commonly associated with intelligent beings” [2]. Over the past decade, particularly in the last 2–3 years, the world has witnessed a transformative surge across nearly every field, driven by advancements in generative AI – a specific type of AI that focuses on generating new content (e.g., text, images, code) based on patterns learned from existing data. The impact spans education, finance, business, healthcare, life sciences, and beyond. Data scientists are among those scientists intimately developing and evaluating AI systems with rigor. By way of background, data science is the science of learning from data and involves the methods used for the analysis and processing of data along with new tools to advance those methods [3]. Despite the data scientist’s involvement, there has been surprisingly little focus on how AI can advance the field of data science and assist data scientists in both research and real-world settings. While numerous symposia have explored the diverse intersections of AI with fields like healthcare, business, and education [4–10], few have focused on AI’s role in the data scientist’s workflow. There are enormous opportunities in data management, analysis, and even study design, where AI may be leveraged. Caution is needed as changes in the workflow can threaten rigor and cause further mistrust of the public in science.

On December 3, 2024, the Stanford Quantitative Sciences Unit co-hosted its inaugural symposium with Stanford Data Science to explore how AI can be integrated thoughtfully into data science workflows in a symposium series entitled *AI for Data Science*. With over 150 in-person attendees, the symposium brought together thought leaders including data science educators, experts in biostatistics, epidemiology, health policy, informatics, and public health to discuss evolving tools, methods, and ethical implications. It aimed to foster collaboration, drive innovation, and identify the specific needs, gaps, opportunities, and challenges for data scientists and their workflows in the AI era. This paper aims to summarize major takeaways from those discussions and propose an agenda for future action and research.

## Methods

### Format

The one-day symposium included remarks from leadership, a keynote address, and three panel discussions on the following topics:

1. Challenges and solutions for integrating AI into the data scientist’s workflow
2. Training and education of current and next generation of data scientists in the era of AI
3. AI for public health to illustrate challenges for data scientists in a real-world setting

### Speakers

Experts from academia, industry, and the public health sector were invited based on their expertise and real-world experience (Table 1).

**Table 1.** Speakers, roles, and job titles

Role	Name	Job Title	Link to Professional Profile
Keynote Speaker	Michael Pencina, PhD	Chief Data Scientist for Duke Health, Vice Dean for Data Science, Director of Duke AI Health, Professor of Biostatistics and Bioinformatics, Duke University School of Medicine	https://medschool.duke.edu/personnel/michael-pencina-phd
Moderator (Panel 1)	Manisha Desai, PhD	Associate Dean for Quantitative and Data Sciences, Director of the Quantitative Sciences Unit, Kim and Ping Li Professor of Medicine and Biomedical Data Science, and by courtesy, of Epidemiology and Population Health, Stanford School of Medicine	https://profiles.stanford.edu/manisha-desai
Panelist (Panel 1)	Nigam Shah, MBBS, PhD	Chief Data Scientist for Stanford Health Care, Associate Dean, Professor of Medicine and Biomedical Data Science, Stanford School of Medicine	https://profiles.stanford.edu/nigam-shah
Panelist (Panel 1)	Jade Benjamin-Chung, MPH, PhD	Assistant Professor of Epidemiology and Population Health, Stanford School of Medicine	https://profiles.stanford.edu/jadebc
Panelist (Panel 1)	Sara Singer, MBA, PhD	Professor of Health Policy and Medicine, Stanford School of Medicine, by courtesy of Organizational Behavior, Stanford Graduate School of Business, and by courtesy, Senior Fellow, Freeman Spogli Institute for International Studies, Stanford University	https://profiles.stanford.edu/sara-singer
Panelist (Panel 1)	James Zou, PhD	Associate Professor of Biomedical Data Science, Stanford School of Medicine, and by courtesy, of Computer Science and Electrical Engineering, Stanford University School of Engineering	https://profiles.stanford.edu/james-zou
Moderator (Panel 2)	Mark A. Musen, MD, PhD	Director of the Stanford Center for Biomedical Informatics Research, Stanford Medicine Professor of Biomedical Informatics Research, Professor of Medicine and of Biomedical Data Science, Stanford School of Medicine	https://profiles.stanford.edu/mark-musen
Panelist (Panel 2)	Bryan Bunning	PhD Student in Biomedical Informatics, Stanford School of Medicine	https://profiles.stanford.edu/bryan-bunning
Panelist (Panel 2)	Laurence Baker, PhD	Josephine Knotts Knowles Professor of Human Biology, Professor of Health Policy, Stanford School of Medicine, Senior Fellow at the Stanford Institute for Economic Policy Research	https://profiles.stanford.edu/laurence-baker
Panelist (Panel 2)	Eleni Linos, MD, MPH, DrPH	Associate Dean for Research, Director of the Stanford Center for Digital Health, Ben Davenport and Lucy Zhang Endowed Professor of Medicine, Professor of Dermatology, Stanford School of Medicine	https://profiles.stanford.edu/eleni-linos
Panelist (Panel 2)	Steven Goodman, MD, MHS, PhD	Associate Dean for Clinical and Translational Research, Professor of Epidemiology and Population Health, of Medicine and, by courtesy, of Health Policy, Stanford School of Medicine	https://profiles.stanford.edu/steven-goodman
Moderator (Panel 3)	Melissa Bondy, PhD	Chair of the Department of Epidemiology and Population Health, Stanford Medicine Discovery Professor of Epidemiology and Population Health and, by courtesy, of Pediatrics, Stanford School of Medicine	https://profiles.stanford.edu/melissa-bondy
Panelist (Panel 3)	John Auerbach, MBA	Senior Vice President, Public Health, ICF, Former Director of Intergovernmental and Strategic Affairs at CDC	https://www.icf.com/company/about/our-people/a/auerbach-john
Panelist (Panel 3)	Ivor Horn, MD, MPH	Former Chief Health Equity Officer at Google, Pediatrician, and Health Services Researcher	https://www.linkedin.com/in/drivorhorn/
Panelist (Panel 3)	Michelle Williams, ScD	Professor of Epidemiology and Population Health, Stanford School of Medicine	https://profiles.stanford.edu/327959
Special Advisor	Lee Sanders, MD, MPH	Division Chief of General Pediatrics, Professor of Pediatrics and Health Policy, and by courtesy of Epidemiology and Population Health, Stanford School of Medicine	https://profiles.stanford.edu/lee-sanders

### Audience

Participants included students, trainees, educators, faculty, and the general public.

## Summarization of talks and panel discussions

### 1. Introduction: the promise and the threat of generative AI to the data scientist’s workflow (Manisha Desai)

Dr Manisha Desai introduced the promises and challenges of AI through illustration of tools including HyperWrite for refining a research question and ChatGPT4.0 for deriving a statistical analysis plan. A recent poll of Dr Desai’s team, the Quantitative Sciences Unit, demonstrated that only a small percentage (<15%) were currently engaging with AI when conducting their work, and that for those who did, they used it for: communication (e.g., explaining models to collaborators), coding, administrative tasks, and for developing statistical analysis plans.

#### Major takeaways

- While there has been increased usage of AI tools in the workflow, this has been done largely without evaluations of how it helps.
- The illustration of HyperWrite for refining a research question demonstrated that the tool was too general to perform such a specialized task and that a better tool – one that was trained on the right data – would be critical to aid researchers in this task.
- The illustration of ChatGPT for creating a statistical analysis plan similarly demonstrated critical errors that did not follow statistical best practices including issues with multiplicity (or inflating the type I error when drawing inference) and the suggested use of an inappropriate outcome measure.
- While caution must be exercised in the use of such tools, some – like ChatGPT – may offer a *start* to a plan that could be further refined.
- Generally, tools that can be effective for data scientists need to be trained on the right data. The user also needs training in how to engage the tool optimally.
- It is essential to keep humans in the loop when developing both research questions and analytic plans. The best AI-based approaches will find ways to do so that facilitate both human creativity and rigorous science.

### 2. Keynote: Robust Governance as a cornerstone of trustworthy AI (Michael Pencina)

Dr Michael Pencina from Duke University School of Medicine delivered his insights on robust governance as the foundation of building and deploying trustworthy AI.

#### Major takeaways

- Users and developers should be brought together to build trust in AI and its capability.
- New methods for evaluating generative AI are needed with two key points in mind: 1) The standard for evaluating generative AI has been human evaluation, but this is not scalable, and 2) Traditional performance metrics for predictive AI do not apply well to generative AI.
- The lack of best practices and guardrails in applications to healthcare delivery have led to inconsistent implementation and potential biases, which are relevant for the data science context.
- In the context of health, joint efforts are emerging in regulators working with industry partners, non-profit organizations, and general public to create flexible frameworks that emphasize local governance with national standards.
- Existing ethical frameworks, such as the Declaration of Helsinki, can be adapted to apply to AI, noting that basic transparency around AI usage is critical.
- Duke’s approach to integrating AI into the healthcare system is the Algorithm-Based Clinical Decision Support (ABCDS) framework which emphasizes the importance of lifecycle management for AI tools, from use-case identification through registration, evaluation, and monitoring.
- Applications of AI in research need to afford sufficient flexibility to promote innovation.
- Extending ideas to the data science workflow:
  - The workflow includes various stakeholders when addressing data-intensive research.
  - New evaluation methods for AI tools and their applications are needed.
  - There has been growing focus on operational AI and data science to enhance health system efficiency.
  - Existing ethical framework need adaption when applying AI to data science practice; basic transparency should be promoted at each step of the data scientist’s workflow.
  - Consensus best practice or applications of AI in data science will promote data science rather than hinder it.
  - Lifecycle management for AI tools are also applicable to data science models and workflows.
  - As in health, we need to emphasize flexible AI governance as a facilitator to data science practice and innovation, avoiding turning it into “research police.”

### 3. Panel 1: challenges and solutions for integrating AI into the data scientist’s workflow

This panel discussed challenges and solutions for integrating AI into the data scientist’s workflow through the following questions:

- 1: What tools might be considered for the data scientist’s workflow?
- 2: How do we evaluate whether a tool is ready for adoption into the workflow?
- 3: How much error is acceptable in research workflows?
- 4: Does integrating AI affect reproducibility compared to traditional statistics or workflows, and how can we ensure reproducibility when using AI tools?

#### Major takeaways

- AI tools are being adapted for various purposes: communication, coding, reproducibility assistance, statistical analysis plan generation, and the analysis of qualitative studies.
- A range of tools are being used to help with activities such as communication (ChatGPT, Claude, Gemini), data sorting, coding, summarization (GitHub Copilot, Cursor AI, CoLoop), reproducibility assistance such as writing README files and bash scripts to create reproducible workflows, enhancing code documentations (ChatGPT), generating statistical analysis plans (ChatGPT), and generating research ideas (e.g., Virtual Lab).
- New tools are being developed by James Zou in his Virtual Lab to create a novel workflow [11]. Sara Singer’s team is developing tools that will integrate into the qualitative researcher’s analytical workflow [12].
- Another interesting use case includes leveraging AI to more efficiently confirm internal reproducibility prior to publication. For example, there may be one person who codes without AI while another codes with AI. This could help reduce the error rate [13].
- The panel acknowledged that tools should be evaluated for their effectiveness for a particular step in the workflow (e.g., how well does Tool A assist in coding this specific problem?), but more importantly, data scientists should evaluate how a given tool affects their entire workflow holistically (e.g., does it reduce the time needed to generate a final statistical analysis plan?).
- We need to rethink how much error is acceptable with a given tool. In making healthcare decisions, small errors can be critical, while in research, error tolerance may be higher. Specifically, we can imagine specifying a tradeoff between efficiency and the error with which we are comfortable. For example, in the discussion, one of the panelists referred to a traditional method to develop a detailed phenotyping algorithm for Type 2 diabetes that required 1,900 hours and achieved 93% precision and 89% recall. With an AI approach, it was discussed that a classifier may be trained on 50 examples in 2 hours and achieve slightly lower precision (around 2% less) but deliver results far more quickly. The key question is: what are the uses for which we need the 1,900 hours version and what can we do with the 2 hours version?
- The stochastic nature of generative AI poses a unique challenge to demonstrating reproducibility, as results can vary each time. Thus, reproducibility exercises need to be structured in a new way. For example, one idea may be to demonstrate reproducibility in steps – breaking the flow apart into pieces where we expect the answer to be constant (where AI was not used) versus dynamic (where AI may have been leveraged to get to the next step). For the dynamic steps, including details of how AI was engaged will be critical.
- Version control (e.g., of the code we generate, or data set we leverage) – while important in research – becomes critical when AI is integrated into the process, especially as we archive our data, code, and other research materials for reproducibility and replicability purposes.

### 4. Panel 2: training and educating current and next generation of data scientists in the Era of AI

This panel focused on the training and education of data scientists through the following questions,

- 1: Considering the rapid advancements in AI, how should we adapt our training and education approach?
- 2: Should we modify our teaching content?
- 3: With the focus shifted toward high-level AI tools and advanced analytics, are we neglecting foundational skills, and what might this mean for future researchers?
- 4: How can we effectively teach fairness, ethics, and recognizing bias, particularly when addressing sensitive data and mitigating bias in practice?

#### Major takeaways

- Educational approaches must evolve to address and acknowledge the integration of AI into research and practice.
- As students may be more proficient in AI than faculty, training educators to be more effective mentors is crucial.
- AI may lower barriers for entry into the field, but fundamental skills – quantitative and analytical skills, communication, and teamwork skills, ethics, and critical thinking – remain vital for evaluating AI tool’s effectiveness.
- Guidelines for AI application in education can reflect our definition of a good and responsible scientist
- Teaching should embrace AI tools while emphasizing the human element in decision-making and realizing AI’s limitations – like its weakness in identifying research gaps or generating original ideas.
- AI tools can reduce technical burdens, allowing educators and students to focus on foundational concepts and deeper intellectual discussions.
- Rising AI usage among students presents challenges in evaluating academic performance, necessitating the incorporation of oral or in-person examinations to assess students’ true understanding of fundamental concepts.
- Now more than ever, ethical practices and bias reduction must be embedded in every aspect of education, with team science approach playing a key role in improving decision-making and mitigating blind spots.

### 5. Panel 3: AI for public health

Our panel addressed the following questions:

- 1: How can public health agencies navigate regulations and data governance challenges to ensure ethical use of AI technologies and to build public trust?
- 2: How can AI unintentionally exacerbate existing health disparities if equity isn’t prioritized in the development of these models?
- 3: How can public health, government, academia, and private sectors collaborate to improve training, build trust, and address policies to prepare for future challenges more effectively and responsibly?

#### Major takeaways

- There are multiple barriers facing the public health sector.
  - The public health sector has long faced limited funding and outdated infrastructure, causing significant barriers to implementing AI technologies despite their potential.
  - The public health sector is not a unified system; local, state, and federal agencies differ widely in technology resources and capacities which leads to uneven adoption and usage of AI across the nation.
- Public health agencies are more likely to adopt AI if the resource threshold for adoption is low and if AI helps solve existing concrete problems or challenges. Early possibilities include use in communication (e.g. translations), administrative task simplification and effective disease surveillance [14–15]. For example, AI can monitor school closures via social media for early warning sign of outbreaks more efficiently than traditional manual means, which frees up skilled individuals for more critical work.
- It is important that AI technology be developed with population heterogeneity in mind [16], recognizing that the pathway to effectiveness of all may require different approaches for different populations.
- Developing AI tools for public health must go beyond surface-level fairness through meaningful collaborations among public health workers, communities, policymakers, and developers, ensuring that AI solutions address root causes of differences in health outcome rather than simply distributing resources equally.
- Building trust and addressing privacy concerns are essential in developing AI tools that improve public health. Trust needs to be built at multiple levels by demonstrating the value and security of AI tools in protecting privacy. Importantly, the tools must be developed with public health workers and the communities they serve in mind and to support – not replace – the public health workers.
- We need to train the public workforce on the use of AI technologies, especially in under-resourced communities.
- To fully unlock AI’s potential in the public health sector, we must leverage public, private, and academic partnerships.

## Conclusion

The AI for Data Science Symposium served as a starting point for exploring the integration of AI into data science. We identified ten important action items (Table 2) for future research. Recommendations emphasize the need for governance, rigorous assessment frameworks, and the development of tools and guidelines that support reproducible AI-based workflows.

**Table 2.** 10 action items

Action Item	Description
1.Develop a flexible facilitating governing framework for prioritizing and integrating AI tools into the data science workflow	The framework for data science should be analogous to frameworks used for health that prioritize tools, with the goal of enhancing and emphasizing innovation, acknowledging the risks are different from those that present in advancing health.
2.Broaden existing guidelines to allow for reproducible workflows for data analysis when AI is integrated	Traditional guidelines for reproducibility may not be applicable to generative AI which is stochastic in nature, necessitating an expansion of traditional guidelines.
3.Develop appropriate guidelines for evaluating different types of AI tools	The unique tradeoff between error and efficiency should be considered in the evaluation process.
4.Develop guidelines for evaluating AI-assisted qualitative analyses for achieving rigor, transparency, and cohesion	While AI increases efficiency, derived themes may contain more noise. Moreover, considering different metrics of evaluation from those in a quantitative setting is important. For example, it may be limiting or misleading to gauge AI output by its ability to exactly reproduce the same output as human investigators. The focus of guidelines should instead be on rigor, transparency, and cohesion.
5.Provide guidelines for evaluating academic progress and achievement in the presence of AI	The growing use of AI may require different methods to evaluate performance and understanding of students and trainees.
6.Ensure curricula for data science are training future trainees to be relevant and critical to research by incorporating key principles around AI development, evaluation, and integration	The emphasis in training should be placed on the principles behind the use of AI rather than specific AI tools, as the field will continue to evolve rapidly.
7.Ensure curricula for the data sciences retain the fundamental principles of the specific data science field	Programs should retain the fundamentals teachings of data science including of study design, statistical inference, probabilistic theory, predictive modeling, resampling methods, coding principles, and other such essentials that enable AI tools to be incorporated responsibly and effectively.
8.Increase AI literacy and competencies among all faculty and trainees in data science	Ongoing training will be essential for current data scientists. Further, new training will need to be developed for emerging data scientists.
9.Enhance the AI literacy across communities that we serve, especially so that trust can be gained by public health officials and by the public themselves	Educating communities on AI is critical, especially for establishing trust within those being served, even if the connection between the scientist’s work and the public is not immediately apparent. Moreover, community feedback can be incorporated to strengthen the effectiveness of the AI tool.
10.Enhance public health literacy in the AI data science community	As data scientists develop and incorporate AI tools into their workflows, understanding the communities they serve will be vital especially for ensuring the relevance and impact of the AI tools developed and adopted.

While all ten action items represent important steps toward advancing AI in data science with rigor and reproducibility, their complexity, resource needs, and dependence on collaboration vary. Some – such as retaining core data science principles in curricula (Item 7) and incorporating AI-related principles into training (Item 6) – can be achieved within existing academic structures, though they require gaining consensus among academic leaders on the core principles. There is no doubt that there will be heterogeneity among institutions in which principles to adopt. Other items, such as developing reproducible workflows for stochastic AI outputs (Item 2) and creating evaluation guidelines for qualitative analyses (Item 4), present greater methodological hurdles. Establishing frameworks for prioritizing and integrating AI tools (Item 1) and developing standards for evaluating different AI tools (Item 3) will require significant cross-disciplinary coordination. Data scientists across subspecialities – for example, biostatisticians trained in evaluation and informaticians trained in large language model development – need to come together to accomplish goals. Items related to literacy – whether among current data scientists (Item 8), trainees (Item 6), or the communities we serve (Items 9 and 10) – are essential for building trust and ensuring relevance, and will require sustained outreach and bidirectional engagement beyond traditional academic settings. The success of the most ambitious items will hinge on broad collaboration, transparency, and shared investment across the data science, AI, and public health communities.
